# Elevated circulating irisin is associated with lower risk of insulin resistance: association and path analyses of obese Chinese adults

**DOI:** 10.1186/s12902-016-0123-9

**Published:** 2016-07-29

**Authors:** Xiulin Shi, Mingzhu Lin, Changqin Liu, Fangsen Xiao, Yongwen Liu, Peiying Huang, Xin Zeng, Bing Yan, Suhuan Liu, Xiaoying Li, Shuyu Yang, Xuejun Li, Zhibin Li

**Affiliations:** 1 Department of Endocrinology and Diabetes, The First Affiliated Hospital, Xiamen University, 55# Zhenhai Road, Xaimen, 361003 China; 2 Department of Nursing, The First Affiliated Hospital, Xiamen University, Xiamen, China; 3 Xiamen Diabetes Institute, The First Affiliated Hospital, Xiamen University, No. 55 Zhenhai Road, Xaimen, 361003 China; 4 Department of Endocrinology, Zhongshan Hospital, Fudan University, Shanghai, China; 5 Epidemiology Research Unit, The First Affiliated Hospital, Xiamen University, Xiamen, China

**Keywords:** Irisin, Insulin resistance, Adiposity, Structural equation modeling

## Abstract

**Background:**

Evidence on the role of irisin in insulin resistance is limited and controversial, and pathways between them remain unknown. We aimed to examine the independent effects of circulating irisin and different adiposity measurements, as well as their potential interactions, on insulin resistance. We also aimed to explore possible pathways among circulating irisin, adiposity, glucose and insulin levels and insulin resistance.

**Methods:**

A cross-sectional study of 1,115 community- living obese Chinese adults, with data collection on clinical characteristics, glucose and lipid metabolic parameters and circulating irisin levels.

**Results:**

Among the 1,115 subjects, 667 (59.8 %) were identified as insulin-resistance, and showed significantly decreased serum irisin than their controls (log-transformed irisin: 1.19 ± 2.34 v.s. 1.46 ± 2.05 ng/ml, *p* = 0.042). With adjustment for potential confounders, elevated circulating irisin was significantly associated with reduced risk of insulin resistance, with adjusted odds ratio per standard deviation increase of irisin of 0.871 (0.765–0.991, *p* = 0.036). As for different adiposity measurements, body fat percentage, but neither BMI nor waist, was significantly associated with increased risk of insulin resistance (OR: 1.152 (1.041–1.275), *p* = 0.006). No significant interaction effect between serum irisin and adiposity on insulin resistance was found. A one pathway model about the relationship between serum irisin and insulin resistance fits well (*χ*^2^ = 44.09, p < 0.001; CFI–0.994; TLI =0.986; and RMSEA = 0.067), and shows that elevated circulating irisin might improve insulin resistance indirectly through lowering fasting insulin levels (standardized path coefficient = −0.046, *p* = 0.032).

**Conclusions:**

Elevated circulating irisin is associated with lower risk of insulin resistance indirectly through lowering fasting insulin.

## Background

Numerous studies have shown that insulin resistance plays an important role in the pathogenesis of metabolic syndrome [1], type 2 diabetes [2] and cardiovascular disease [3], but the mechanisms underlying insulin resistance are not fully understood. Skeletal muscle accounts for the largest organ of the body in non-obese individuals and has been identified to be an important endocrine organ, producing myokines [4]. Irisin, a newly identified myokine, drives brown-fat-like conversion of white adipose tissues, and therefore has been suggested to improve metabolic and glucose homeostasis [5]. In our two previous publications, we found that serum irisin levels were negatively associated with risks of metabolic syndrome [6] and chronic kidney disease [7], in which the mechanisms of associations both included insulin resistance. However, existing evidence on the role of irisin in insulin resistance is limited and controversial. Moreno-Navarrete JM and co-workers firstly reported circulating irisin levels were negatively associated with obesity and insulin resistance in men [8]. Meanwhile Park KH et al. found that irisin was positively associated with fasting glucose and homeostasis model assessment for insulin resistance (HOMA-IR); and that irisin was independently associated with HOMA-IR in multiple linear regression analyses after adjustment for confounders [9]. Moreover, data on the association between irisin and insulin resistance in obese adults are scarce.

Obesity has been conclusively found to be associated with increased risks of insulin resistance, type 2 diabetes, hypertension, cardiovascular disease and cancer [10]. Although body mass index (BMI) has been widely used as the index of obesity, numerous studies have reported that BMI and other measures of adiposity, such as waist circumference, body fat percentage, show different associations with various health conditions [11–13]. Irisin is suggested to be not only a myokine but also an adipokine [14], therefore, effects of different adiposity measurements, as well as their potential interactions with circulating irisin, on insulin resistance should be explored further.

Bostrom et al. reported that expression of the exercise- and PGC1-α*-*induced irisin drives brown fat-like development of white fat and protects diet-induced obesity and diabetes in mouse models [5]. They found that irisin is released into blood from skeletal muscle after proteolysis of the type I membrane protein FNDC5, stimulates uncoupling protein 1 (UCP1) expression, increases total energy expenditure, and then improves glucose tolerance and reduces fasting insulin in animal models [5]. However, there is no evidence available on the pathways about the relationships among serum irisin, adiposity, glucose and insulin levels with insulin resistance in humans, therefore studies are warranted to explore the potential pathways among them, which may be helpful in elucidating the mechanisms of insulin resistance.

In the present study based on baseline examination of the same cohort of 1,115 community-living healthy obese Chinese adults as our two previous publications [6, 7], we firstly aimed to examine the independent effect of circulating irisin levels on insulin resistance. Secondly, different body composition measurements of adiposity, as well as their potential interactions with circulating irisin, on insulin resistance will be tested further. Thirdly, we will explore the possible pathways among circulating irisin, adiposity, glucose, insulin levels and insulin resistance.

## Methods

### Study population

The present study was based on the baseline examination of the same cohort of 1,523 community-living healthy obese Chinese adults as our two previous publications [6, 7]. Details on methods of subject sampling, recruitment and evaluation have been described in these two publications [6, 7]. Briefly, a total of 1,523 subjects aged 40 years or older living in the Lianqian community, Xiamen, China with central obesity (waist circumference greater than 90 cm for men and 80 cm for women) were recruited. Of them, 1,115 (73.2 %) subjects with the complete data on the entire examination were left for further analysis. The study was approved by the Human Research Ethics Committee of the First Affiliated Hospital of Xiamen University (Xiamen, China). Written informed consent was obtained from each participant.

### Measurements

#### Clinical parameters

Anthropometric measurements were obtained using standard protocols and techniques. After removal of shoes and heavy clothing, each subject underwent weight, height and waist circumference measurements, using a calibrated scale. Body mass index (BMI) was calculated as weight in kilograms divided by height in squared meters as a measure of general obesity. Waist circumference was measured at the midpoint between the inferior costal margin and the superior border of the iliac crest on the midaxillary line. Body fat were quantified with the Hologic whole body DXA systems (Hologic Inc., Bedford, MA). Arterial blood pressure was measured with a mercury sphygmomanometer after sitting for at least 15 min. Blood pressure measurements were taken according to the Joint National Committee VII criteria (JNC VII) [15]. Three readings were taken at 5-min intervals. The mean of the three measurements was recorded.

#### Biochemical parameters

All blood samples were obtained after 12-h fasting. Blood and urine samples were refrigerated at −20 °C, transferred and tested in the central laboratory of the First Affiliated Hospital, Xiamen University. 75-g oral glucose tolerance test (OGTT) was conducted for each subject. Plasma glucose, liver enzyme levels, and serum lipid profiles, including triglyceride (TG), total cholesterol (TC), and high-density lipoprotein cholesterol (HDL-C) were determined on a HITACHI 7450 analyzer (HITACHI, Tokyo, Japan). Low-density lipoprotein cholesterol (LDL-C) was calculated by Friedewald’s formula. Fasting plasma glucose concentration (FPG) and 2-h plasma glucose concentration (2-h PG) were measured by the hexokinase method. Serum fasting insulin concentration was measured by electrochemiluminiscence immunoassay (Roche Elecsys Insulin Test, Roche Diagnostics, Mannheim, Germany). Serum irisin concentration was measured using the enzyme-linked immunosorbent assay (ELISA) kits (Aviscera Biosciences, Santa Clara, CA). The sensitivity of the assay was 0.2 ng/ml and the linear range of the standard was 5 to 500 ng/ml. The intra- and inter- assay variations were both less than 10 %. Homeostasis model assessment - insulin resistance (HOMA-IR) was calculated using the formula: fasting serum insulin (mU/L) *fasting plasma glucose (mmol/L) /22.5. And insulin resistance was defined as HOMA-IR ≥ 2.6*10^−6^mol*U/L^2^ [16].

### Statistical analysis

Data were presented as the mean ± standard deviation for continuous variables or number and percentage for categorical variables. Irisin was log-transformed to obtain better approximation of normal distribution. Differences between subjects were analyzed using one way ANOVA for continuous variables and chi-square test for categorical variables.

Multivariable logistic regression was used to calculate adjusted odds ratio (OR) and 95 % confidence intervals (CI) of circulating irisin levels and different adiposity measurements for insulin resistance in different models with adjustment for potential confounders. In model 1, age and sex were adjusted for; in model 2, educational level, smoking and drinking habits and regular physical exercise plus model 1 were adjusted for; in model 3, systolic blood pressure and HDL-C plus model 2 were adjusted for. Different adiposity measurement, such as BMI, waist circumference and body fat percentage, were tested in the multivariable logistic regression models separately. In each of the multivariable logistic regression models, interaction effects between serum irisin and adiposity measurements were tested separately.

Structural equation modeling (SEM) was conducted to test the relationships among serum irisin, adiposity, glucose and insulin levels with insulin resistance. Standardized path coefficients and the significant direct effects are presented. In conducting SEM analyses, satisfactory model fitting was indicated by root mean square error of approximation (RMSEA) ≤0.10 [17] and by comparative fit index (CFI) and Tucker-Lewis fit index (TLI) ≥0.90 [18].

Structural equation modeling (SEM) was conducted using *lavaan (0.5–19)* package in R3.2.2 [19]; all the other analyses were performed using Stata14.0 (StatCorp, College Station, TX). All p-values were two-sided and *p*-value <0.05 was considered statistically significant.

## Results

Of the 1,115 participants, 766 (68.7 %) were female. The mean age (±SD) of women and men were 53.1 (±7.1) years and 53.3 (±7.6) years (*p* = 0.702), respectively [6, 7]. The overall prevalence rate (95 % confidence interval (CI)) of insulin resistance was 59.8 % (56.9–62.7 %).

### Demographic and clinical characteristics stratified by insulin resistance

Differences in demographics, life style habits, body composition measurements and clinical characteristics of subjects stratified by insulin resistance are presented in Table 1. Generally, when compared with controls, subjects with insulin resistance had significantly higher levels of systolic and diastolic blood pressure, triglyceride, total cholesterol, fasting plasma glucose (FPG), 2-h PG (OGTT), fasting insulin, HbA1c and lower levels of HDL-C. Subjects with insulin resistance showed significantly higher adiposity levels than those without (BMI: 28.7 ± 3.3 v.s. 26.6 ± 2.4 kg/m^2^; waist: 94.8 ± 7.5 v.s. 91.9 ± 6.0 cm; body fat percentage: 35.6 ± 7.0 % v.s. 33.3 ± 6.1 %; all *p*-values < 0.001). Subjects with insulin resistance also showed significantly decreased serum irisin levels than their controls (log-transformed irisin levels: 1.19 ± 2.34 v.s. 1.46 ± 2.05 ng/ml, *p* = 0.042). There was no significant difference of age, gender, educational level and lifestyle habits between subjects with and without insulin resistance.

**Table 1:** Demographic, lifestyle and clinical characteristics of subjects by insulin resistance

	Insulin Resistance		
Variables	No	Yes	*P* value
Demographics			
*N* (%)	448 (40.1 %)	667 (59.8 %)	
Sex			0.765
Female (*n*, %)	305 (39.8 %)	461 (60.2 %)	
Male (*n*, %)	143 (41.0 %)	206 (59.0 %)	
Age (years)	52.8 ± 7.2	53.4 ± 7.2	0.191
Education categories, (*n*, %)			0.591
Illiteracy	112 (37.1 %)	190 (62.9 %)	
Elementary school	138 (41.8 %)	192 (58.2 %)	
Middle school	101 (39.5 %)	155 (60.5 %)	
High school	58 (40.8 %)	84 (59.2 %)	
College or above	39 (45.9 %)	46 (54.1 %)	
Life style habits			
Ever smoking (*n*, %)	126 (42.7 %)	169 (57.3 %)	0.334
Ever drinking (*n*, %)	60 (46.2 %)	70 (53.8 %)	0.194
Regular physical exercise (*n*, %)	159 (44.0 %)	202 (56.0 %)	0.079
Adiposity measurements			
BMI (kg/m^2^)	26.6 ± 2.4	28.1 ± 3.3	<0.001^***^
Waist circumference (cm)	91.9 ± 6.0	94.8 ± 7.5	<0.001^***^
Body fat (%)	33.3 ± 6.1	35.6 ± 7.0	<0.001^***^
Clinical characteristics			
Systolic blood pressure (mmHg)	128.9 ± 16.8	137.4 ± 17.7	<0.001^***^
Diastolic blood pressure (mmHg)	77.1 ± 10.4	81.7 ± 10.8	<0.001^***^
Triglyceride (mmol/L)	1.52 ± 0.95	2.11 ± 1.41	<0.001^***^
Total cholesterol (mmol/L)	5.76 ± 1.00	5.94 ± 1.13	<0.001^***^
HDL-cholesterol (mmol/L)	1.43 ± 0.29	1.32 ± 0.29	<0.001^***^
LDL-cholesterol (mmol/L)	3.65 ± 0.93	3.66 ± 1.07	0.878
Fasting glucose (mmol/L)	5.52 ± 0.58	6.59 ± 2.11	<0.001^***^
2-h PG (OGTT) (mmol/L)	7.40 ± 2.28	10.16 ± 4.64	<0.001^***^
HbA1c	5.91 ± 0.48	6.40 ± 1.25	<0.001^***^
Fasting insulin (mU/L)	7.64 ± 1.92	16.15 ± 6.73	<0.001^***^
HOMA-IR (*10^−6^mol*U*L^−2^)	1.87 ± 0.48	4.67 ± 2.44	<0.001^***^
Irisin-log transformed (ng/ml)^a^	1.46 ± 2.05	1.19 ± 2.34	0.042*

All percentages are row percentage; except for percentages, all values are mean ± SD

*Abbreviations*: *BMI* body mass index, *HDL* high-density lipoprotein, *HOMA* homeostasis model assessment, *IR* insulin resistance index, *LDL* low-density lipoprotein cholesterol, *OGTT* oral glucose tolerance test, *PG* plasma glucose

*p < 0.05, ***p < 0.001

^a^Irisin was log-transformed to obtain better approximation of normal distribution

### Associations between circulating irisin levels, adiposity measurements and insulin resistance

Adjusted odds ratios (ORs) with associated 95 % confidence interval (CI) of circulating irisin levels and adiposity measurements for insulin resistance are shown in Table 2. In model 1 with adjustment for sex and age, elevated serum irisin levels were significantly associated with reduced risk of insulin resistance, the adjusted OR (95 % CI) of per standard deviation (SD) increase of serum irisin was 0.878 (0.776–0.993, *p* = 0.039). When each of the adiposity measurements was added into the model separately, increasing levels of adiposity, such as BMI, waist circumference and body fat percentage, were all significantly associated with increased risks of insulin resistance. But when all the three body composition measures were added into the model simultaneously, only body fat percentage, but neither BMI nor waist, was significantly associated with increased risk of insulin resistance (OR (95 % CI): 1.202 (1.092–1.323), *p* < 0.001). And there was no significant interaction effect between serum irisin and adiposity measurements on insulin resistance.

**Table 2:** Adjusted odds ratios (ORs) with associated 95 % confidence interval (CI) for insulin resistance

	Insulin Resistance		
Variables	OR	95 % CI	*P* value
Model 1			
Serum irisin^a^	0.878	0.776–0.993	0.039*
Adiposity measurements			
BMI (kg/m^2^)	1.212	1.155–1.272	<0.001^***^
Waist circumference (cm)	1.079	1.056–1.102	<0.001^***^
Body fat (%)	1.174	1.132–1.217	<0.001^***^
Interaction effect			
Serum irisin* Body fat (%)			0.640
Model 2			
Serum irisin^a^	0.875	0.773–0.989	0.034*
Adiposity measurements			
BMI (kg/m^2^)	1.211	1.154–1.271	<0.001^***^
Waist circumference (cm)	1.079	1.056–1.103	<0.001^***^
Body fat (%)	1.174	1.131–1.217	<0.001^***^
Interaction effect			
Serum irisin * Body fat (%)			0.624
Model 3			
Serum irisin^a^	0.871	0.765–0.991	0.036*
Adiposity measurements			
BMI (kg/m^2^)	1.157	1.101–1.217	<0.001^***^
Waist circumference (cm)	1.063	1.039–1.086	<0.001^***^
Body fat (%)	1.132	1.090–1.176	<0.001^***^
Interaction effect			
Serum irisin* Body fat (%)			0.817

Model 1 was adjusted for sex and age

Model 2 was further adjusted for educational level, ever smoking, ever drinking and regular physical exercise

Model 3 was further adjusted for systolic blood pressure and HDL

* *p* < 0.05, ^***^ *p* < 0.001

^a^OR and 95%CI was expressed as per SD increase of log transformed serum irisin

In model 2 (additional adjustments for educational level, smoking and drinking habits and regular physical exercise) and model 3 (further adjustments for systolic blood pressure and HDL-C), the results were quite similar to those in model 1. In model 3, the adjusted OR(95 % CI) of per SD increase of serum irisin for insulin resistance was 0.871 (0.765–0.991, *p* = 0.039). When all three adiposity measurements were added into the model 3 simultaneously, only body fat percentage was significantly associated with increased risk of insulin resistance (OR (95 % CI): 1.152 (1.041–1.275), *p* = 0.006). When serum irisin and body fat percentage were added into model 3 simultaneously, the effect of circulating irisin on insulin resistance was attenuated into marginally significant (OR (95 % CI): 0.879 (0.770–1.003), *p* = .055), but the effect of body fat percentage on insulin resistance showed little change. And interaction effect between serum irisin and adiposity measurement on insulin resistance was not statistically significant.

### Possible pathways for serum irisin leading to insulin resistance

**Fig. 1:**
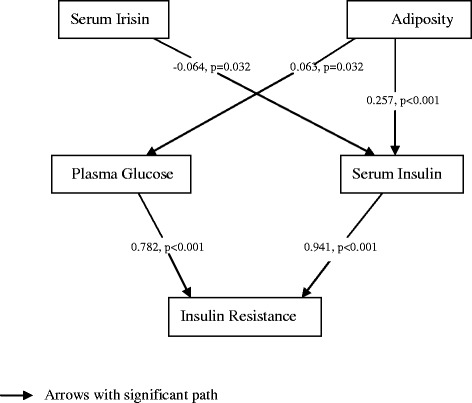
Structural equation modeling path diagrams for associations among serum irisin, adiposity, plasma glucose, serum insulin and insulin resistance

SEM analysis about the relationships among serum irisin, adiposity, glucose, insulin and insulin resistance shows that a one pathway model fits well (*χ*^2^ = 44.09, *p* < 0.001; CFI–0.994; TLI =0.986; and RMSEA = 0.067) (Fig. 1). The standardized path coefficients in the path diagrams indicate that serum irisin might lower the risk of insulin resistance indirectly. Increased circulating irisin levels might lower fasting insulin levels (standardized path coefficient = −0.046, *p* = 0.032), which in turn improves insulin resistance. Adiposity (body fat percentage) is associated with increased fasting insulin levels and FPG (standardized path coefficient: 0.257 (*p* < 0.001) and 0.063 (*p* = 0.032), respectively), which then increases the risk of insulin resistance. No significant effect of serum irisin on adiposity or FPG was observed.

## Discussion

The present study demonstrated that circulating irisin levels were significantly decreased in obese Chinese adults with insulin resistance than their controls and that elevated circulating irisin were independently associated with reduced risk of insulin resistance. Although obesity has been conclusively found to be associated with insulin resistance, we found that body fat percentage, but neither BMI nor waist circumference, was independently associated with insulin resistance after adjusting for potential confounders. Our novel finding on possible pathways of insulin resistance shows that elevated irisin might decrease fasting insulin level and then reduce the risk of insulin resistance indirectly, and adiposity might increase both fasting insulin and FPG levels and therefore induce insulin resistance.

Myokines secreted by skeletal muscle which has been considered as the largest endocrine organ have been suggested to play a key role in glucose and lipid metabolism, thus contributing to energy homeostasis and pathogenesis of insulin resistance related health conditions, such as metabolic syndrome and diabetes [20]. Irisin, a newly identified myokine, was reported to increase total energy expenditure, improve glucose tolerance and reduce fasting insulin, drive brown fat-like development of white fat and then protect diet-induced obesity and diabetes in mouse models in 2012 [5]. After that, great interests have been attracted to explore the role of irisin in humans diseases even therapeutic potentials on obesity and diabetes [21, 22]. As for the relationship between irisin and insulin resistance in humans, available evidence has been controversial. Moreno-Navarrete firstly reported circulating irisin levels were negatively associated with obesity and insulin resistance in men [6]. Negative association between circulating irisin levels and HOMA-IR were also reported by Hu W and Huth C et al. [23, 24]. In high-fat diet induced obese mice, Yang et al. reported that irisin secretion decreased which contributed to muscle insulin resistance [25]. Contrastingly, Park et al. found that irisin was positively associated with fasting glucose and HOMA-IR in a sample of 151 subjects; and that irisin was independently associated with HOMA-IR and increased risk of metabolic syndrome in multiple regression analyses after adjustment for confounders. They discussed that either increased secretion by adipose/muscle tissue and/or a compensatory increase of irisin to overcome an underlying irisin resistance in these subjects [9]. Similar findings were also reported by others [26–28]. Reinehr et al. found that irisin levels were the highest in obese children with impaired glucose tolerance, followed by obese children with normal glucose tolerance, and were the lowest in normal weight children. After adjusted for confounders in a multiple regression analysis, they found that baseline irisin was significantly associated with HOMA-IR and concluded that irisin concentrations were positively related to insulin resistance and parameters of metabolic syndrome, which might be because obese children with features of metabolic syndrome are in an irisin-resistant state [26]. It should be noted that the sample sizes of all of the studies above are quite small. In the present study with a sample size of 1,115 obese adults, we found that circulating irisin levels were significantly decreased in those with insulin resistance than those without, and furthermore, elevated circulating irisin were independently associated with reduced risk of insulin resistance after adjusting for potential confounders. The controversy about the effect of irisin on insulin resistance should be tested further, especially by the designed prospective cohort studies in different populations.

Bostrom et al. reported that the exercise- and PGC1-α*-* induced irisin has been proposed to act as a hormone on subcutaneous white cells, increasing energy expenditure in mouse models by means of a program of brown-fat-like development, which is not yet fully elucidated [5, 29]. Irisin has been suggested to increase total energy expenditure, improve glucose tolerance and reduce fasting insulin, therefore induce improvement of glucose homeostasis, insulin resistance, and obesity-related health conditions [30, 31]. However, no evidence is available on the possible pathways among serum irisin, adiposity, glucose, insulin levels and insulin resistance in humans. An novel finding of the present study on possible pathways for insulin resistance is that elevated circulating irisin levels improve insulin resistance indirectly through decreasing fasting insulin. We previously reported that fasting insulin was negatively associated with serum irisin level in a stepwise multivariable linear regression analysis [6]. The negative effect of circulating irisin on fasting insulin in obese adults in the present study was consistent with Bostrom’s report in mice [5]. But the mechanisms by which irisin decreases fasting insulin should be clarified in future. We did not find any direct effect of serum irisin on either fasting glucose or adiposity, which was also in line with many previous studies [8, 32].

Obesity has been conclusively found to be associated with increased risks of insulin resistance, but BMI has been widely used as the index of obesity in these studies. Since numerous studies have reported that different measurements of adiposity, such as BMI, waist circumference and body fat percentage, show different associations with various health conditions [11–13], therefore effects of different adiposity measurements on insulin resistance should be explored. The present study found that body fat percentage, but neither BMI nor waist circumference, was independently associated with insulin resistance, which implicated that adiposity per se, rather than body weight or body shape, is independently associated with increased risk of insulin resistance. Therefore, decreasing body fat percentage should be emphasized when dealing with weight reduction for those obese subjects.

We should be cautious during interpretation of the present findings due to the following limitations. One limitation is that HOMA-IR calculated with fasting glucose and fasting insulin levels rather than hyperinsulinemic euglycemic clamp, which remains the “gold standard” for accurately determining insulin resistance, were used in the present study. The second limitation is that subjects were not randomly sampled from their living communities and were all central obese adults, so we cannot extrapolate our findings to the non-obese adults. The third limitation is that we are not certain about the temporal sequence between serum irisin and insulin resistance because of the cross-sectional study design. Therefore, our results should be confirmed in the designed prospective cohort studies or intervention studies in future. Excess adiposity has been reported to be associated with increased circulating adipokine concentrations, such as leptin, and leptin has been recently found to down-regulate FNDC5 expression in the adipose tissue [33, 34]. In the present study, subjects with insulin resistance showed higher body fat percentage than their controls, therefore, the inhibitory effect of leptin on adipose FNDC5 expression may also explain the decreased circulating irisin in subjects with insulin resistant. Therefore, the fourth limitation is that we did not measure other cytokines, such as leptin, adiponectin, IL-6 and TNF-α, which may be related to serum irisin level and may confound the association between irisin and insulin resistance. We must acknowledge that the detection of circulating irisin remains largely controversial, since the commercial antibodies and ELISA assays reveal prominent cross-reactivity with non-specific proteins in human and animal sera and ELISA assays have not been widely accepted as accurate and reproducible [35]. Although the intra and inter assay variations in the present study were both less than 10 %, detection of circulating irisin could be another limitation of the present study. On the other hand, these limitations do not diminish the value of our study. Firstly, our study has a relatively large sample size of obese adults. And we have also adjusted for much more potential confounders than previous studies. For example, few previous studies adjusted for physical activity, body composition measurements, which were important confounding factors of serum irisin. Last but not the least, we are probably the first to report the possible pathways among circulating irisin, adiposity, glucose, insulin levels and insulin resistance. By using structural equation modeling, we found that elevated circulating irisin levels improve insulin resistance indirectly through lowering fasting insulin, which may be helpful to elucidate the mechanisms of insulin resistance.

## Conclusions

In conclusion, based on the baseline examination of the same cohort of 1,115 community-living healthy obese Chinese adults as our two previous publications [6, 7], the present study further demonstrated that circulating irisin levels were significantly decreased in those with insulin resistance compared to their controls. And elevated circulating irisin improves insulin resistance indirectly through lowering fasting insulin, although the underlying mechanisms are not yet fully understood. Furthermore, body fat percentage, rather than body weight or body shape, is independently associated with increased risk of insulin resistance. Therefore, decreasing body fat percentage should be emphasized for obese subjects to prevent incidence of insulin resistance related health conditions. Future studies about effects and possible pathways of irisin on insulin resistance with the designed prospective cohort studies and intervention studies in the general adults are warranted therefore.

## Abbreviations

BMI, body mass index; CFI, comparative fit index; CI, confidence interval; ELISA, enzyme-linked immunosorbent assay; FPG, fasting plasma glucose; HbA1c, hemoglobin A1c; HDL-C, high density lipoprotein cholesterol; HOMA, homoeostasis model assessment; IR, insulin resistance; LDL-C, low density lipoprotein cholesterol; OGTT, oral glucose tolerance test; OR, odds ratio; RMSEA, root mean square error of approximation; SEM, structural equation modeling; TC, total cholesterol; TG, triglyceride; TLI, Tucker-Lewis fit index
